# Renal transplantation: An update

**DOI:** 10.4103/0970-1591.33723

**Published:** 2007

**Authors:** Aneesh Srivastava

**Affiliations:** Division of Urology and Renal Transplantation, Sanjay Gandhi Post Graduate Institute of Medical Sciences, Lucknow - 226 014, UP, India

The magnitude of end-stage renal disease (ESRD) in India still remains largely undetermined. It is stated that around 80,000 patients are added annually to the ESRD pool and need some kind of renal replacement therapy. Renal transplantation as a specialty is now well established in India and close to 3600 transplants are being performed annually. This symposium on renal transplantation is aimed at various issues which are pertinent to the Indian scenario.

In view of the large number of transplants being done, the need for a national registry was immensely felt since a long time. Dr. Sunil Shroff under the auspices of the Indian Society of Organ Transplantation has given a definite shape to this task with mammoth dimensions and this will go a long way in improving the existing practices of transplantation in the country. Current statistical figures reveal that data from many prominent centers have yet to be incorporated. The importance of providing necessary data to the national transplant registry cannot be overemphasized.

Establishing a secure vascular access is the immediate priority once chronic renal failure has been established with potential for progression to ESRD. The brachiocephalic fistula is an alternative option if the distal fistula either fails or the veins are thrombosed. Authors from SGPGI have suggested the use of the radial artery about 2 cm below the elbow instead of doing venous anastomosis to the brachial artery. The advantages seem to outweigh the disadvantages.

Safer surgeons and anesthesia with more effective immuosuppression has led to much less restrictive selection criteria for transplantation than in the past. Yet the need for a careful assessment cannot be undermined in view of the high prevalence of co-morbid diseases in the recipients. The American Society of transplant physicians has prepared an excellent review of the assessment process.[1] In this issue, Dr. Modi has discussed the relevant aspects of pre-transplant recipient workup in detail.

There has always been a wide gap between the demand and supply of the organs. Use of suboptimal kidneys from living and deceased donors and non-heart-beating donors is an attempt to narrow the margins of this disparity. There are existing British and European guidelines on this issue. Dr. Ganesh Gopalkrishnan has discussed the issue threadbare and given very useful guidelines from the Indian perspective. An alternative option to expand the living donor pool for recipients who have a willing donor but cannot donate directly because of a positive cross-match or ABO blood group incompatibility is paired donation. Paired donation is where incompatible donor recipient pairs swap donors to achieve transplantation that does require extraordinary recipient immune modulation, in essence avoiding the incompatibility. I personally feel that the concept of a national donor kidney exchange may yield more instantaneous results as compared to promoting the deceased donor program.

Laparoscopic donor nephrectomy has reduced disincentives for living renal donors. Should it be the transperitoneal or retroperitoneal approach is best decided by the individual surgeon depending upon his level of comfort. Inherent problems with obtaining a reasonable length of renal vein on the right side have deterred the surgeons from retrieval of right kidney. The left-sided kidneys even with multiple vessels are therefore being preferably retrieved in order to give the advantage of minimal invasion to the patient. Our group has one of the largest experiences with transperitoneal left-sided nephrectomy and the pertinent issues have been addressed here.

After live donor nephrectomy, cooling is done soon after retrieval and the kidney is therefore subjected to minimal warm ischemia. Simple ice cooling along with an intra-arterial flush with a cold preserving solution helps to preserve the function. Several preservation solutions like UW and Euro Collins etc. are available. Ringer lactate is used in this country in many centers basically for economic reasons. Authors from CMC Vellore in a well-designed study have proven that Euro Collins is a better perfusion fluid than Ringer lactate. This kind of study brings a very pertinent issue forward in the scenario of a developing country. We all modify our clinical practice depending upon the geographical location and the availability of local resources and feel that our results are as good as what are described in the literature without actually having any objective documentation. The centers with bulk in a particular area should identify such practices and subject them to a controlled trial in order to substantiate them scientifically.

Anomalies of the lower urinary tract (congenital or acquired) account for 20-30% of pediatric renal failure cases.[1] Treatment of children with ESRD, especially those with significant bladder dysfunction is difficult. A high-pressure and low-capacity bladder is a major risk factor for a transplanted kidney. These patients are usually managed initially with a combination of CIC and drugs. Patients with neurogenic bladder would usually need an augmentation cystoplasty. Dr. Mahesh Desai has addressed this issue and given some useful guidelines.

History repeats itself. We are all aware that Lord Ganesh is cited as an example of xenotransplantation from the Indian mythology. For the last few decades a number of attempts have been made to transplant kidneys from non-human primates to patients. Recently, the pig has been seen as the animal of choice and future utilization of pig organs for transplant may revolutionize transplantation. Dr. Carl G. Groth, an eminent Professor of Transplantation surgery from Sweden is involved with pioneering research in this field. In his comprehensive article he has given an insight into the work done so far in this field along with futuristic projections.

I would like to thank all the contributors and the editorial team of the IJU to have given me this opportunity.
